# Factors associated with current smoking in COPD patients:A cross-sectional study from the Eastern Black Sea region of Turkey

**DOI:** 10.18332/tid/90665

**Published:** 2018-05-22

**Authors:** Dilek Karadogan, Ozgur Onal, Deniz Say Sahin, Yalcın Kanbay

**Affiliations:** 1 Department of Chest Diseases, School of Medicine, Recep Tayyip Erdoğğan Üniversitesi, Rize, Turkey; 2 Department of Public Health, School of Medicine, Süleyman Demirel University, Isparta, Turkey; 3 Department of Social Services, Faculty of Economics and Administrative Sciences, Mehmet Akif Ersoy University, Burdur, Turkey; 4 Department of Psychiatric Nursing, School of Health Science, Çoruh University, Artvin, Turkey

**Keywords:** smoking, COPD, current smokers, former smokers, factors

## Abstract

**INTRODUCTION:**

Even though smoking is a major reason for the development and progression of chronic obstructive pulmonary disease (COPD)-and quitting smoking is the only way to stop its progression-a significant number of smokers still continue to smoke after being diagnosed with COPD. The aim of this study is to compare the clinical and demographic characteristics of COPD patients who are current and former smokers and to find factors associated with their current smoking status.

**METHODS:**

For this study, data were collected between June 2015 and August 2016; COPD patients who had been regularly visiting Hopa State Hospital’s outpatient clinic over the last year or longer were included. Their demographic, clinical and functional data were recorded. Patients completed a pulmonary function test, six-minute walk test (6-MWT), COPD assessment test (CAT), and modified Medical Research Council (mMRC) dyspnea scale. Comparisons were then made according to their smoking status.

**RESULTS:**

In total 100 patients were included in the study; with a mean age of 63.4±10.7 years and mostly males (94%). Regarding smoking status, 49% were current smokers and 51% were former smokers. Multivariate logistic regression analysis revealed that current smoking was negatively associated with age (odds ratio, OR=0.93, 95% confidence interval, CI=0.88–0.96) and Global Initiative for Chronic Obstructive Lung Disease (GOLD) stage (OR=0.32, 95% CI=0.13– 0.79), and was positively associated with six-minute walk distance (OR =1.005, 95% CI=1.001–1.009) and CAT score (OR=1.07, 95% CI=1.009–1.13).

**CONCLUSIONS:**

Nearly half of the COPD patients in the study continued smoking even after having been diagnosed with COPD. The younger patients, with better lung function, better exercise capacity and poor quality of life were associated with current smoking.

## INTRODUCTION

Chronic Obstructive Pulmonary Disease (COPD) is a major preventable and treatable public health challenge and the fourth-leading cause of death in the world. COPD is the result of cumulative exposures to particles over decades. Globally, cigarette smoking is the most commonly encountered risk factor for COPD; smoking cessation is the only effective treatment to stop the progression of COPD^1^. In a previous study, it has been determined that mild-to moderate-COPD patients who stopped smoking experienced an improvement in forced expiratory volume in 1 second (FEV_1_) in the year after quitting^2^. However, even though smoking is the major cause of the development and progression of COPD-and quitting smoking is the only way to stop its progression-a significant number of smokers with COPD still continue to smoke after being diagnosed. In previous studies, the prevalence of current smokers was found to be higher in COPD patients compared to healthy groups^3-5^. Some of the reasons that have been posited to explain the higher smoking prevalence among COPD patients include: higher physical dependency upon nicotine, longer exposure time to tobacco, higher depression rate, and a higher rate of patients with lower education level. All of these factors have also been found to have a role in COPD patients’ less successful quitting interventions^6^. However, in other studies, smokers with airway obstruction have been found to be more likely to quit smoking^7^.

There are also qualitative studies in the literature that evaluate the reasons why COPD patients continue to smoke^8-10^. Few studies, however, directly compare the demographic and functional characteristics of current and former smokers who are COPD patients^11,12^. Therefore, we intended to evaluate the differences between these two groups. Our study’s contribution to the literature is a better understanding of the differences between smoker and former smoker COPD patients, in the interest of discovering a more effective path to encourage COPD patients to cease smoking after diagnosis.

## METHODS

### Study population and design

Hopa State Hospital is a secondary health-care unit in the eastern Black Sea region of Turkey. A pulmonologist, a hospital staff member, follows up COPD patients in an outpatient clinic at regular intervals-every 3 to 6 months. In this COPD outpatient clinic, each patient has a file containing their sociodemographic characteristics, disease history, physical examination results, laboratory tests results, pulmonary function test (PFT), 6-minute walk test (6-MWT) records, modified Medical Research Council dyspnea scale (mMRC), and COPD assessment test (CAT) scores. There were 117 patients having regular follow-ups in the COPD outpatient clinic at the time of the study. During visits, appropriate patients who wanted to join the study were included after giving written informed consent. Inclusion criteria were: a) patients who had received a COPD diagnosis at least one year ago according to GOLD guidelines1 and had regularly attended their control visits at the COPD outpatient clinic during the last year; b) patients who were in a stable period of the illness^1^; c) patients who could undergo the PFT, 6-MWT, mMRC and CAT tests; and d) patients who were current smokers at the time of their initial COPD diagnosis.

The exclusion criteria were: a) patients who were evaluated to be in the COPD exacerbation period according to Global Initiative for Chronic Obstructive Lung Disease (GOLD) guidelines1; b) patients who did not want to join the study; c) patients who could not complete the PFT and the questionnaires; d) patients with cognitive disorders due to diseases like dementia, Parkinson’s disease or schizophrenia; e) patients with a walking disability due to any orthopedic or neurological diseases; and f) patients with higher scores in the Beck anxiety scale (≥8) and/or Beck depression scale (≥10).

Each of the patients who had quit smoking after the COPD diagnosis had done it on their own without using smoking cessation medications. All of them reported that they had received advice from their doctor to quit and had been informed about the harmful effects of smoking on their lung health.

### Ethics approval, consent to participate and data collection

Before starting the study, ethics approval (3 July 2015) was received from the Ethics Committee of the Rectorship, Artvin Çoruh University. Permission for the study was also obtained from Artvin Government Hospital’s general secretary. Written informed consent forms of the participants were obtained by the first author of the study. Afterwards, data collection started in June 2015 and ended August 2016. In the first part of the study, the researchers used face-to-face interviewing of patients by the pulmonologist to obtain information about their age, sex, education level, current work status, smoking status, comorbid diseases, COPD diagnosis time, number of exacerbations and hospitalizations due to COPD in the last year, answers to mMRC dyspnea scale^13^, and CAT^14^.

In the second part of the research, participants’ height and weight were measured wearing light clothes, no hat or shoes, by the PFT technician nurse^15,16^. The 6-MWT^17^ and PFT were performed by the same trained nurse.

### Questionnaire

The CAT is a short, specific quality of life (QoL) questionnaire for measuring the impact of COPD on a patient’s well-being and daily life. It consists of eight items evaluating the frequency of cough, phlegm, chest tightness, breathless level at exercise, sleep status, and energy status, with scores ranging from 0 to 5 (0–no impairment, 5–greatest impairment). An overall score is calculated by adding the score from each item, with total scores ranging from 0 to 40, the higher scores indicating more severe health status impairment or a poorer control of COPD^14^. We used a Turkish version of the CAT that has already been translated and validated for use^18^.

### Functional assessment

PFT: The lung function test was performed in a seated position using a Spirovit SP-260 spirometer (Schiller Medical, Moscow, Russia) consistent with American Thoracic Society (ATS)/European Respiratory Society (ERS) guidelines. Forced vital capacity (FVC), FEV_1_ and FEV_1_/FVC rate were recorded. Lung function test parameters were expressed as a percentage of the predicted values for age, height, body weight, and sex^15,16^.

6-MWD: This was performed according to ATS guidelines^17^. Patients were asked to walk quickly along a 30 metre level corridor for 6 minutes, and the total distance walked was recorded in metres. The test was carried out twice with a 30-minute interval. The best value of the two tests was used for statistical analysis. Heart rate and oxygen saturation were recorded during the 6-MWD using a pulse oximeter (PlusMED, plus-50DL, Turkey).

### Information on potential confounders

Smoking status was recorded as former smoker or current smoker. Anyone who had smoked more than 100 cigarettes in their lifetime and smoked in the last 28 days was classified as a current smoker; someone who had smoked more than 100 cigarettes in their lifetime but had not smoked in the last 28 days was classified as a former smoker^19^. Number of pack-years (packs smoked per day × years as a smoker) was also recorded. A history of comorbid disease was defined as a positive answer to questions regarding physician-diagnosed: diabetes, hypertension, cerebrovascular disease, pulmonary disease, ischemic heart disease, and other diseases. Employment status was asked of each patient, and at the time of data analysis categorized as retired or working actively. Body mass index (BMI) was calculated as weight (kg) divided by height (m) squared. COPD exacerbations and hospital-treated COPD exacerbations in the last year were included in patient interviews, and also checked through the recorded hospital database. GOLD stage1-4 and GOLD category (A, B, C, D) were recorded according to GOLD guidelines^1^.

### Statistical analysis

Data were analyzed using Statistical Package for the Social Sciences version 20. First, descriptive analysis (mean, proportions) was performed for each dependent variable. Categorical variables were described using their absolute and relative frequencies, while quantitative variables were described by the mean and standard deviation. To evaluate the relationship between independent and dependent categorical variables, Pearson’s chi-squared test or the Fisher exact test was used; for numerical variables an independent sample t-test was used. The associations were considered significant at p<0.05. Odds ratio (OR) univariates were calculated by logistic regression to evaluate the different risks contemplated in the study, including all demographic, clinical and quality-of-life variables. After backward analysis, the various adjusted ORs were calculated using multivariate logistic regression, and only statistically significant associations are shown in Table 1.

**Table 1:** Factors associated with current smoking in univariate and multivariate analysis. Hopa State Hospital, 2015–2016 (n=100 )

	*Univariate Analysis*			*Multivariate Analysis*		
	*OR*	*95% CI*	*p*	*OR*	*95% CI*	*p*
Age (per 1-year increment)	0.93	0.89–0.97	0.001	0.93	0.88–0.96	0.009
Employment status (working actively)	3.21	1.28–8.06	0.013			
Hospitalization in last year (per 1 number increment)	0.66	0.48–0.90	0.01			
FEV1% (per 1% increase)	1.03	1.00–1.06	0.009			
FVC% (per 1% increase)	1.03	1.01–1.05	0.004			
GOLD stage (per 1 stage increment)	0.42	0.21–0.81	0.01	0.32	0.13–0.79	0.013
6-MWD (per 1 m increase)	1.00	1.00–1.00	0.001	1.005	1.001–1.009	0.02
MMRC (per 1 stage increase)	0.63	0.45–0.90	0.01			
CAT (per 1 stage increase)	1.00	0.96–1.04	0.88	1.07	1.009–1.13	0.02
COPD category (per 1 category increase)	0.27	0.12–0.64	0.003			
Disease duration (per 1 year increase)	0.92	0.86–0.98	0.02			
Number of COPD medications (per 1 medication increase)	0.63	0.43–0.94	0.02			

Values are expressed as odds ratio (OR) and confidence interval (CI). Multivariate analysis was controlled for age, employment status, hospitalization number, FEV1% predicted, FVC% predicted, GOLD stage, 6-MWD, mMRC score, CAT score, COPD category, disease duration and number of COPD medications.

## RESULTS

In total 100 patients were included in the study; mean age of the patients was 63.4±10.7 years and 94% of patients were male. Most of the patients had graduated from primary school (72%), were retired (71%), were in GOLD stage 2 or 3 (36% and 54%, respectively), had an mMRC dyspnea score of 2 or higher (67%), and had a high (≥10) CAT score (87%). Of the participants, 49% were current smokers. Detailed characteristics of the patients and the comparisons according to their smoking status are seen in Table 2.

**Table 2:** Characteristics of patients according to their cigarette smoking status; Hopa State Hospital, 2015–2016 (n=100 )

	*All patients 100*	*Current smokers 49*	*Former smokers 51*
			
**Age (mean±SD)**	63.4±10.7 (min 40–max 85)	59.6±9.1*	67.0±10.9
**Sex**			
Male (n, %)	94 (94%)	47 (95.9%)	47 (92.2%)
Female	6 (6%)	2 (4.1%)	4 (7.8%)
**Education level**			
0	2 (2%)	1 (2.04%)	1 (1.96%)
5 year primary schooling	72 (72%)	32 (65.3%)	40 (78.4%)
8 years secondary schooling	8 (8%)	4 (8.1%)	4 (7.8%)
11 years high schooling	13 (13%)	8 (16.3%)	5 (9.8%)
15 years university	5 (5%)	4 (8.1%)	1 (1.96%)
**Work status**			
Actively working	29 (29%)	20 (40.8%)*	9 (17.6%)
Retired	71 (71%)	29 (59.1%)	42 (82.3%)
**Smoking (pack/year)**	44.2±16.2 (min 20–max 100)	43.5±13.9	44.9±18.2
**COPD diagnosis age (mean)**	56.1±10.3	54.3±8.7	57.9±11.4
**Disease duration (mean±SD)**	7.2±7.7	5.30±5.54*	9.09±8.98
**BMI (kg/m²) (mean±SD)**	26.4±5.9	26.1±5.74	26.6±6.16
**Number of exacerbations in last 1 year (mean±SD)**	2.52±2.53	2.18±1.99	2.84±2.94
0	14 (14%)	8 (16.3%)	6 (11.7%)
1	30 (30%)	14 (28.5%)	16 (31.3%)
2–5	46 (46%)	23 (46.9%)	23 (45.0%)
>5	10 (10%)	4 (8.1%)	6 (11.7%)
**Number of hospital-treated exacerbations (emergency+admission)**	1.14±1.68	0.67±1.04*	1.58±2.04
0	51 (51%)	31 (63.2%)*	20 (39.2%)
≥1	49 (49%)	18 (36.7%)	31 (60.7%)
**FEV_1_ % (mean±SD)**	49.3±15.7	53.6±17.0*	45.1±13.3
**FVC%**	62.4±21.3	70.5±23.3*	55.5±16.8
**FEV_1_/FVC**	60.0±8.26	59.9±9.66	60.0±6.97
**GOLD stage**			
1	5 (5%)	4 (8.1%)	1 (1.9%)
2	36 (36%)	23 (46.9%)	13 (25.4%)
3	54 (54%)	20 (40.8%)	34 (66.6%)*
4	5 (5%)	2 (4.08%)	3 (5.8%)
**6–MWD (mean±SD) in metres**	335.5±120	377.2±122.1*	289.9±101.1
**MMRC dyspnea score (Mean±SD)**	2.2±1.2	1.91±1.18	2.54±1.17
<2	33 (33%)	19 (38.7%)	14 (27.4%)
≥2	67 (67%)	30 (61.2%)	37 (72.5%)
**CAT (mean±SD)**	19.4±9.1	19.5±9.22	19.3±9.15
<10	15 (15%)	9 (19.5%)	6 (11.7%)
≥10	85 (85%)	40 (81.6%)	45 (88.2%)
**COPD category**			
A	11 (11%)	8 (16.3%)*	3 (5.8%)
B	30 (30%)	19 (38.7%)	11 (21.5%)
C	5 (5%)	0	5 (9.8%)
D	54 (54%)	22 (44.8%)	32 (62.7%)
**Number of patients with LTOT**	10 (10%)	2 (4.08%)	8 (15.6%)
**Number of COPD medications (mean±SD)**	3.09±1.07	2.84±1.10*	3.33±0.99
**Number of comorbidities**	0.46±0.64	0.35±0.56	0.57±0.70
	Column percentages are given		

Values are expressed as mean (SD) and frequency (%).

* p-value<0.05 compares current smoker and former smoker patients.

CAT: COPD assessment test; FEV1: forced expiratory volume in 1 second; FVC: forced vital capacity; SD: standard deviation; BMI: body mass index; 6-MWD: 6-min walking distance (m); mMRC: modified Medical Research Council dyspnea scale; LTOT: long-term oxygen therapy.

In univariate analysis, current smoking was negatively associated with age (p=0.001), GOLD stage (p=0.01), COPD category (p=0.003), number of hospitalizations (p=0.01), mMRC dyspnea score (p=0.01), disease duration (p=0.02), and number of COPD medications (p=0.02). Current smoking was positively associated with FEV_1_ % predicted (p=0.009), FVC % predicted (p=0.004), and 6-MWD (p=0.001). However, in multivariate analysis, current smoking was negatively associated with age (OR=0.93, 95% CI=0.88–0.96, p=0.009) and GOLD stage (OR=0.32, 95% CI=0.13–0.79, p=0.013), and was positively associated with 6-MWD (OR=1.005, 95% CI=1.001–1.009, p=0.02) and CAT score (OR=1.07, 95% CI=1.009–1.13, p=0.02) (Table 1).

## DISCUSSION

Our findings showed that among COPD patients, current smokers were more likely to be younger, have better lung function, walk a longer 6-MWD, and conversely, have worse quality of life, detected by the higher scores in the CAT, compared to former smokers.

Previous studies around the world have shown that among COPD patients, current smokers’ prevalence is between 33.6% and 47.2%^3-5,11,12,20^. Moreover, current smoking prevalence has been found to be higher among COPD patients compared to healthy individuals in previous international studies^3,5^. Similarly, in this study we found COPD patients’ current smoking rate to be 49% (50% among males and 33.3% among females), while the general recent smoking prevalence among individuals older than 40 years in Turkey is approximately 40% among men and 13% among women^21^. While there has been a decrease in the population’s current smoking prevalence compared to previous years^21^, according to our findings there was no decrease among COPD patients’ current smoking prevalence compared with previous national studies^22,23^. Turkish COPD patients’ smoking prevalence is estimated to be approximately 60%.

One interesting indicator was that while lung function and mobility/endurance were relatively unaffected in COPD patients who continued to smoke, potentially because of their youth or shorter smoking time, quality of life was still significantly lower-yet not low enough to have an apparent impact on motivation to quit. It is essential to find solutions for successful cessation interventions for patients in this group, because smokers with COPD are shown to be less successful in quitting^24^. Though there are various reasons cited for this trend^3,25^, in previous studies of COPD patients those who were able to quit were found to be older^26^ and have a further advanced disease^11^. Furthermore, patients who are beginning to lose the ability to take part in certain activities are more likely to quit, most likely in an effort to regain those abilities^4^. In other words, current smokers are living with less severe COPD-related activity limitations, and so their perception of COPD may be different from those with more severe symptoms. At diagnosis, more intensive education should be given to COPD patients, not only about inhaler use but also about outcomes and systemic effects of COPD, and the importance of quitting smoking and avoiding secondhand smoke exposure as well. At that point, brief interventions including our results could be an effective way to encourage smoking cessation in newly diagnosed, young COPD patients at the earlier stages of the disease^27^. Particularly, pointing out the increased respiratory symptoms and lowered QoL-even compared to older, more severe COPD patients who no longer smoke-could be the key. Patients should be supported in their quit attempts not only with motivational counselling but also with changes in tobacco control policies, such as covering smoking cessation interventions and approved SC medications under health insurance, as recommended in a previous guidance^28^. COPD patients demonstrate proven cost-effectiveness of free SC interventions, and they need support to overcome nicotine withdrawal symptoms and other challenges is smoking cessation^29-31^.

Most qualitative studies evaluating reasons for continued smoking in COPD patients found that cigarettes are regarded like friends. For other patients, quitting smoking after diagnosis was considered useless and too late to make a difference, even though they were aware that their COPD was caused and exacerbated by smoking^9,32^. In this study, we even verified that all of the patients were aware that the main reason for the development of their illness was smoking, because they had been followed-up with and educated about the illness in the COPD outpatient’s clinic. However, we did not obtain qualitative information about their reasoning for continuing to smoke. To our knowledge there is no qualitative study that evaluates Turkish COPD patients’ smoking status. Learning the reasons why patients believe they still can continue to smoke would be important to discern and so better manage their quit attempts.

Our study showed that higher CAT scores were associated with current smoking. Similarly, Cheruvu et al.^12^ found their current smoker COPD patients’ health-related quality of life to be worse than former smokers, regardless of their younger mean age or disease severity. CAT score can change or improve via many factors, including smoking cessation and pulmonary rehabilitation^33^. In a recent study, a group of smoker COPD patients’ CAT scores decreased from 18.9±7.3 to 8.1±6.1 six months after quitting smoking^34^. This is an important point for smoker COPD patients to understand, and that they can expect an improvement in QoL after quitting smoking, which should motivate them in their quit attempts. Explaining and comparing QoL should be an integral part of COPD diagnosis and education.

Only 100 participants were found who were both current patients at the clinic and met the inclusion criteria. The clinic/hospital was located in a small town. Furthermore, many patients did follow-ups with their own internal medicine specialists or family doctors. As a result, not all of the local COPD patients apply to the pulmonology outpatient clinic. That may explain the low number of the study population, as the methodology of face-to-face interviews required convenience sampling. We did not use any biological confirmation of smoking status, instead grouping according to patient report. This is one of the limitations of the study. But it has been shown that smokers with COPD do report their smoking status reliably^35^. The evaluation of comorbid diseases also depended on the patients’ self-report; we did not do additional tests to evaluate the comorbid diseases. This may have lowered the prevalence of comorbid disease in the study; it has been reported that comorbidity prevalence and detection rates can be variable due to the research method used and style of questioning^36^. We asked our patients: ‘Do you have any other diseases, such as cardiac disease, diabetes, hypertension?’. Therefore, most of the reported comorbidities included were from those systems. Other less severe diseases may not have been reported by the patient. Another limitation is that we could not be sure whether former smokers quit when their disease was at its early stages. It was not possible to evaluate former smokers’ functional capacity and COPD clinical status at the time of their successful quitting intervention.

## CONCLUSIONS

According to this study, nearly half of COPD patients continued to smoke even after they had been diagnosed with COPD. Among them, those who were younger, were at the early stages of the disease, and had better performance status on the walk test, were more likely to continue to smoke. To determine the reasons behind these results, qualitative studies, which are extremely rare in our country, are needed to evaluate the underlying reasons of COPD patients’ smoking status. The fourth factor, lower QoL, may not explain reasons for continued smoking but instead be a valuable reference to discourage it after diagnosis.
